# Juvenile habitat partitioning and relative productivity in allochronically isolated sockeye salmon (*Oncorhynchus nerka*)

**DOI:** 10.1002/ece3.55

**Published:** 2011-12

**Authors:** EK Fillatre Miller, IR Bradbury, DD Heath

**Affiliations:** 1 Great Lakes Institute for Environmental Research and the Department of Biological Sciences, University of Windsor OntarioCanada; 3 Fisheries and Oceans CanadaScience Branch, Canada

**Keywords:** Allochronic, genotype assignment, habitat partitioning, microsatellite, productivity, sockeye

## Abstract

Allochronic divergence, like spatial isolation, may contribute to population diversity and adaptation, however the challenges for tracking habitat utilization in shared environments are far greater. Adult Klukshu River (Yukon, Canada) sockeye salmon, *Oncorhynchus nerka*, return as genetically distinct “early” and “late” runs. Early and late adult spawning populations (1999 and 2000) and their subsequent fry (sampled at 7 sites in 2000 and at 8 sites in 2001 throughout Klukshu Lake and River) were genotyped at eight microsatellite loci. Bayesian assignment was used to determine the spatial distribution of early versus late fry; although intermixed, the distribution of fry significantly differed in Klukshu Lake and in the Klukshu River in 2001, based on crosstab analyses. Late-run fry predominated in Klukshu Lake at all sites, while early-run fry were most common in the north and south of Klukshu Lake and in Klukshu River. Early-run spawners had significantly higher relative productivity (early life survival) than late-run fish (2.9 times more fry produced per early-run adult in 2000, and 9.2 times more in 2001). This study demonstrates spatial habitat partitioning and differences in the contribution of allochronically isolated populations to fry abundance, and highlights annual variability that likely contributes to recruitment variation.

## Introduction

Understanding the distribution of biological diversity in space and time, thought central to the stability and persistence of populations, remains an ongoing challenge for conservation efforts. Increasing evidence supports a link between both species and population diversity (e.g., Tilman 1996; Schindler et al. 2010) and the stability and persistence of ecosystems and species. In exploited species, failure to identify critical intraspecific diversity risks the overexploitation and extinction of small and vulnerable populations. This loss of critical intraspecific diversity may threaten the ability of species to respond to changing environmental conditions, increase the variance in ecosystem dynamics, and ultimately affect the stability and persistence of populations and fisheries (Hilborn et al. 2003; Schindler et al. 2010).

Intraspecific diversity has been well documented in natural populations (e.g., Taylor and Bentzen 1993; Vamosi and Schluter 1999; Lecomte and Dodson 2005), most often associated with habitat discontinuities, environmental gradients (e.g., Bradbury et al. 2010), or trophic specialization (Schoener 1974; McPhail 1993; Rogers and Bernatchez 2007). In seasonal environments, population isolation may also occur temporally, and examples of allochronic isolation have been identified in insects (Santos et al. 2007), plants (Ellis et al. 2006), fish (Hendry and Day 2005), and marine invertebrates (Tomaiuolo et al. 2007). Adaptive diversity along seasonal gradients is likely to be important to a species’ response to changing environmental conditions, and hence represent a significant component of its adaptive portfolio. However, for mobile and highly dispersive species, such as marine or anadromous fish, conservation of this “portfolio” requires an understanding of the spatial and temporal distribution of populations across their life history.

Allochronic divergence in salmonid fishes where stocks return to natal streams at different times within a year is exhibited in many Pacific salmon species, including pink (*Oncorhynchus gorbuscha*), steelhead (*Oncorhynchus mykiss*), chum (*Oncorhynchus keta*), coho (*Oncorhynchus kisutch*), and Chinook (*Oncorhynchus tshawytscha*) (see Hendry and Day 2005 for review). In sockeye salmon (*Oncorhynchus nerka*), several studies have reported significant genetic divergence among runs entering a spawning area at intervals of weeks to months (e.g., Woody et al. 2000; Fillatre et al. 2003; Hendry et al. 2004). Fillatre et al. (2003) observed that the genetic divergence between two sockeye salmon runs in the Klukshu River, Yukon, was substantial (*F_ST_* = 0.023) and stable over 7 years of sampling (1994–2000). The two Klukshu sockeye runs differ in their date of return to the mouth of the Klukshu River on their migration run by over 30 days (Fillatre et al. 2003), and since early-run fish have been observed spent and dying within 2 weeks of the beginning of the early-run (Fillatre et al. 2003), it is reasonable to conclude that the two runs are at least partially temporally reproductively isolated. Differences in reproductive timing expose populations to differing environmental conditions and may be linked to variation in habitat usage and/or thermal tolerance, both of which likely represent critical determinants of biodiversity. The main objective of this work is to examine the consequences of divergence and allochronic isolation in sockeye from the Klukshu River, Yukon for the spatial distribution of juveniles. Specifically, we address three main questions: (1) Where are the sockeye salmon fry found in the Klushu River system?; (2) Are the early- and late-run offspring spatially intermixed or do they partition their rearing habitat due to either adult or juvenile habitat preferences?; and, (3) What is the relative productivity of the early run versus late run? These questions were addressed using a combination of intensive habitat sampling and the application of molecular genetic assignment methods. Habitat partitioning as examined here may reflect the consequences of both active habitat choice as well as choice of adult spawning timing or location. We report that juvenile early- and late-run sockeye salmon in the Klukshu River system are not completely intermixed, may utilize different rearing habitat, and display substantial differences in productivity.

## Methods and Materials

### Study system

**Figure 1:**
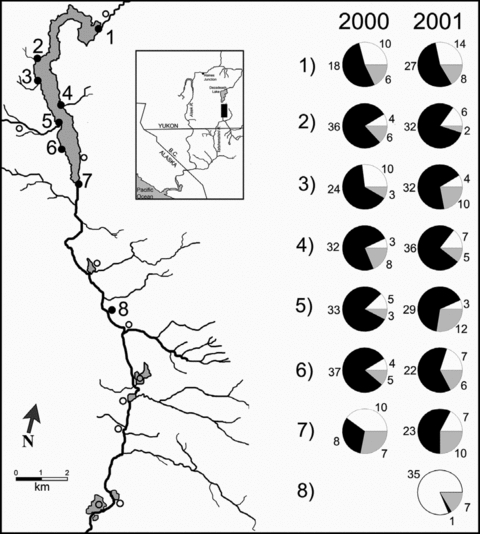
Map showing sockeye salmon fry sampling sites in the Klukshu River system (inset map shows the location of the study system). Open circles on the map depict sites sampled where no fry were captured. The eight filled circles on the map show the Klukshu Lake and River sites included in the multilocus genotype assignment analysis to identify each fry as having had an early-run parent (open pie segment, at right), late-run parent (black pie segment, at right), or failed assignment (grey pie segment, at right). Failed assignment fry are those that had intermediate rank-based assignment scores.

Klukshu River and Klukshu Lake are located approximately 400 km southwest of Whitehorse, Yukon (60° N and 137° W; Fig. 1). Klukshu River supports sockeye salmon returns ranging from 5,000 to 30,000 fish annually. Sockeye salmon migrating to Klukshu River return in two pulses, with peak returns before (“early” run) and after August 15th (“late” run; Fillatre et al. 2003). Early- and late-run spawning occurs mainly in Klukshu River (which drains the lake) and Klukshu Lake, based on radio telemetry (Petkovich 1999), although additional spawning sites are possible. The Klukshu system also supports populations of Chinook salmon (*O. tshawytscha*), coho salmon (*O. kisutch*), whitefish (*Coregonus clupeaformis*), and sculpins (*Cottus sp*.). The lake is characterized by a variety of fish habitat: at the northern end of the lake (site 1; Fig. 1), the bottom substrate is soft and muddy, with year-round inflow from Little Klukshu River. Steep rocky shores predominate further south, and no suitable juvenile salmon habitat (i.e., no littoral zone or freshwater inflow or upwelling) is available until site 2 (Fig. 1). The northwest of the lake (sites 2 and 3; Fig. 1) is characterized by intermittent water runoff and medium to large gravel substrate, while the east shore (site 4; Fig. 1) has intermittent flow, shallow habitat, and fine gravel substrate. The southwest area of the lake (sites 5 & 6; Fig. 1) has intermittent flow, possibly significant ground water runoff, small to medium gravel substrate, and narrow littoral zones with rapid increases in depth. At the southern end of the lake (Fig. 1) shallow water with grassy vegetation and soft substrate is dominant, while the mouth of Klukshu Lake (site 7; Fig. 1) has small pebble substrate.

### Field sampling

#### Juvenile sampling

The Klukshu system was intensively surveyed for sockeye salmon fry in 2 years; 2000 and 2001 (Fig. 1). In 2000, Klukshu River, Klukshu Lake, and associated tributaries were intensively sampled to identify where the sockeye fry were rearing. The choices were narrowed to eight sampling sites (Fig. 1) through exploratory seine sets: if no fry were captured after three to five seine sets, the site was deemed to be not used as juvenile rearing habitat. The selected sites were sampled in July and August in 2000 (*N*[total] = 325; range by site = 28–54), and again in June and July in 2001 (*N*[total] = 391; range by site = 36–52) using minnow traps and seine nets. Minnow traps were set in slow to medium moving river, and in shallow lake (0.30–1.5 m) habitats, and collected and checked after 12–24 hours. Seine nets were also used within pools and slow to medium moving water. Klukshu sockeye fry remain in the freshwater rearing habitat for 2 years, after which they migrate to the salt water. We sampled age one (recently hatched) juvenile fish (fry) in 2000 and 2001, and caudal fin clips were collected. Following sampling, sockeye fry were released unharmed and fin samples were preserved in 95% ethanol for subsequent DNA extraction. The possibility of misidentifying 1-year-old fish as young-of-the-year fish was minimal due to large size and coloration differences (Age 0 fork length = 38 ± 4 [SE] mm; Age 1 fork length = 65 ± 7 [SE] mm).

#### Adult sampling

All adult sockeye salmon returning to the Klukshu system pass through a counting weir where fish are manually counted, sampled for scales and/or fin clips before being released above the weir. For this study, adult sockeye salmon tissue samples (fin clips) were obtained from the peaks of the early- and late-run Klukshu River sockeye salmon in 1999 (*N*[early] = 49; *N*[late] = 39) and 2000 (*N*[early] = 30; *N*[late] = 57) (see Fillatre et al. 2003 for details) and represent the parental population of the 1-year-old fry we sampled in 2000 and 2001, respectively.

### Microsatellite genotyping and data analysis

Sockeye salmon fry DNA was isolated using WIZARD genomic purification kits (Madison, WI, USA). The adult DNA extraction protocol was a modified proteinase K and phenol:chloroform:isoamyl alcohol (PCI) technique, as described in Fillatre et al. (2003). Fry and adult sockeye were genotyped at eight highly variable microsatellite loci. Microsatellite polymerase chain reactions (PCRs) were performed as described in Fillatre et al. (2003), using the same microsatellite loci (Table 1). PCR products from the fry and parental fish were analyzed for molecular size (±0.5 bp) using an automated DNA analyzer; allele sizes were determined using the manufacture's software (Visible Genetics, Toronto, Canada) and verified by manual allele size identification. Approximately 5% of all PCR reactions were replicated to test for repeatability (97% of the alleles agreed across the two replicates, departures were typically due to single repeat size differences).

**Table 1:** Number of alleles (*A*), sample sizes (*N*), observed and expected heterozygosity (*H_O_* and *H*_E_), and *F*_IS_ at eight microsatellite loci for Klukshu River sockeye salmon fry sampled at 7 and 8 sites in 2000 and 2001, respectively. Significant deviations from Hardy–Weinberg equilibrium are denoted by grey shading of the boldface *F*_IS_ values

		2000							2001							
			
Locus		Site 1	Site 2	Site 3	Site 4	Site 5	Site 6	Site 7	Site 1	Site 2	Site 3	Site 4	Site 5	Site 6	Site 7	Site 8
Oneu2	*N*	50	52	43	50	47	45	25	49	49	48	52	48	36	49	47
	*A*	11	10	8	10	10	7	7	10	10	9	10	9	9	10	8
	*H_O_*	0.58	0.65	0.60	0.60	0.55	0.51	0.76	0.67	0.59	0.81	0.62	0.58	0.58	0.80	0.75
	*H_E_*	0.68	0.73	0.67	0.67	0.65	0.62	0.73	0.58	0.71	0.69	0.58	0.62	0.59	0.75	0.78
	*F_IS_*	0.14	0.08	0.08	0.09	0.14	0.16	–0.07	–0.16	0.17	–0.18	–0.06	0.03	0.02	–0.07	0.04
Oneu8	*N*	49	52	43	50	54	46	27	52	49	41	52	49	36	49	49
	*A*	7	9	7	7	9	9	21	9	8	8	7	9	9	10	8
	*H_O_*	0.73	0.75	0.87	0.76	0.85	0.89	0.89	0.75	0.73	0.88	0.75	0.69	0.83	0.74	0.96
	*H_E_*	0.77	0.76	0.76	0.72	0.74	0.74	0.79	0.78	0.73	0.77	0.75	0.70	0.79	0.79	0.81
	*F_IS_*	0.05	0.00	–0.17	–0.06	–0.15	–0.21	–0.13	0.04	0.00	–0.16	–0.01	0.02	–0.05	0.07	–0.18
Ots3	*N*	38	36	30	37	45	34	21	51	46	46	51	48	36	48	45
	*A*	5	6	6	6	6	6	5	5	5	4	6	6	7	6	6
	*H_O_*	0.55	0.71	0.64	0.51	0.47	0.79	0.48	0.53	0.59	0.57	0.73	0.56	0.56	0.63	0.40
	*H_E_*	0.60	0.64	0.65	0.66	0.60	0.70	0.69	0.62	0.67	0.60	0.66	0.66	0.68	0.63	0.42
	*F_IS_*	0.06	–0.17	0.02	0.22	0.20	–0.13	0.29	0.13	0.13	0.02	–0.11	0.15	0.17	0.01	–0.01
Oneu18	*N*	50	52	43	50	54	48	28	52	50	50	52	50	36	49	50
	*A*	8	5	8	6	8	5	8	5	8	8	7	4	5	5	5
	*H_O_*	0.68	0.79	0.62	0.72	0.64	0.68	0.79	0.65	0.80	0.70	0.62	0.70	0.75	0.69	0.46
	*H_E_*	0.83	0.79	0.77	0.80	0.83	0.76	0.83	0.70	0.77	0.76	0.71	0.71	0.77	0.74	0.50
	*F_IS_*	0.18	–0.02	0.15	0.10	0.21	0.09	0.05	0.06	–0.04	0.07	0.14	0.01	0.01	0.06	0.05
Usat60	*N*	50	52	43	50	54	47	28	52	50	51	52	50	36	50	50
	*A*	9	7	5	5	6	5	7	5	7	5	5	4	4	6	3
	*H_O_*	0.82	0.75	0.69	0.70	0.79	0.79	0.54	0.73	0.66	0.77	0.75	0.66	0.69	0.66	0.70
	*H_E_*	0.82	0.67	0.69	0.70	0.74	0.71	0.76	0.72	0.69	0.68	0.67	0.64	0.73	0.68	0.53
	*F_IS_*	0.01	–0.13	0.04	0.00	–0.08	–0.09	0.30	–0.04	0.03	–0.13	–0.11	–0.03	0.05	0.03	–0.32
One108	*N*	49	52	43	50	54	47	27	52	49	50	52	50	36	50	49
	*A*	17	6	14	12	17	12	11	13	15	12	12	11	9	10	10
	*H_O_*	0.86	0.87	0.83	0.74	0.77	0.83	0.70	0.75	0.83	0.76	0.81	0.76	0.86	0.70	0.80
	*H_E_*	0.93	0.86	0.87	0.86	0.89	0.88	0.88	0.85	0.86	0.83	0.85	0.84	0.82	0.83	0.79
	*F_IS_*	0.07	0.01	0.01	0.13	0.12	0.07	0.20	0.12	0.03	0.08	0.04	0.09	–0.05	0.16	0.00
Ssa85	*N*	50	52	43	50	54	46	28	52	50	48	52	50	36	50	50
	*A*	12	12	12	14	13	13	11	16	17	17	15	17	13	19	16
	*H_O_*	0.82	0.73	0.87	0.82	0.81	0.80	0.79	0.85	0.82	0.83	0.85	0.72	0.72	0.88	0.88
	*H_E_*	0.85	0.86	0.88	0.85	0.86	0.83	0.84	0.88	0.89	0.89	0.88	0.89	0.83	0.90	0.90
	*F_IS_*	0.04	0.16	0.05	0.03	0.05	0.02	0.07	0.03	0.07	0.06	0.03	0.19	0.13	0.02	0.01
One115	*N*	50	51	43	50	54	47	27	52	50	50	52	50	36	50	50
	*A*	29	12	18	19	22	18	16	27	28	28	30	26	19	28	23
	*H_O_*	0.96	0.90	0.85	0.88	0.93	0.96	0.89	0.92	0.94	0.94	0.98	0.84	0.97	0.98	0.90
	*H_E_*	0.96	0.92	0.93	0.92	0.92	0.92	0.94	0.95	0.94	0.95	0.94	0.92	0.93	0.95	0.89
	*F_IS_*	0.00	0.04	0.05	0.04	0.00	–0.05	0.05	0.02	0.00	0.01	–0.04	0.08	–0.05	–0.03	–0.01

An exact test for goodness of fit to Hardy–Weinberg equilibrium was conducted for adult early- and late-run fish in 1999 and 2000, using Arlequin version 3.11 (Excoffier et al. 2005), and adjusted for significance using sequential Bonferroni correction. Population structure was evaluated for the 1999 and 2000 adults by calculating pairwise *F_ST_* using TFPGA 1.3 (Miller 1997). An exact test for goodness of fit to Hardy–Weinberg equilibrium was conducted at all loci for the fry samples in both years (2000 and 2001) using the Monte Carlo method (20,000 permutations). The results of the Hardy–Weinberg test were adjusted for significance using the sequential Bonferroni correction (Rice 1989).

We used genotype assignment to assign sockeye salmon fry (unknown) to their early- and late-run parental (source) population for the 2 years of adult-fry paired sampling (e.g., 1999–2000 and 2000–2001). The genotype assignment used a two-step process in Gene Class 2.0 (Piry et al. 2004). First, we used the partial Bayesian method of Rannala and Mountain (1997) to exclude fish having both assignment likelihoods below a 10% threshold. Next, fish were scored as “early” or “late” run fry using the rank-based assignment method with the criterion that the likelihood score of assignment must exceed 80% for the individual to be successfully assigned. One possible source of error for this analysis would be if the early- and late-run adult sockeye were misclassified due to errant run timing to the mouth of the Klukshu River (where they were sampled). However, the early run and late run were well differentiated temporally in both years with the DNA sampling separated by more than 4 weeks (Fillatre et al. 2003). The mean straying rates between the early- and late-run adults were estimated to be less than 4% in 1999 and less than 7% in 2000 (Fillatre et al. 2003), making it unlikely that parental misclassification contributed substantially to the assignment error.

Once the fry had been assigned to early or late populations, we tested for global differences in the spatial distribution of early- and late-run fry across the seven lake sampling sites (Fig. 1; sites 1–7), using two-way crosstab chi-square analyses in 2000 and in 2001. We then tested for individual site deviations from the total early- and late-run fry proportions using two-way crosstab comparisons within each year separately. Since sockeye fry were only captured at site 8 (Klukshu River, Fig. 1) in 2001, we excluded that site from our analyses. Finally, crosstab analyses was used to test for significant year-to-year (2000–2001) differences in the proportion of early- and late-run fry at each site separately.

The relative productivity (i.e., production of fry) of each run was determined by comparing the proportion of adults (based on numbers at the counting weir) to the proportion of fry recruits (based on the assignment results) in both the early run and late run for the replicated adult-fry sample groups. Since our estimates of the absolute numbers of early- and late-run fry are based on a subsample of the fry present at each sample site, we used proportional comparisons of adult and juvenile abundance. Thus, for example, if the relative productivity of the early- and late-run adult sockeye were equal, we would expect our random sample of fry to generate the same proportion of early- to late-run juveniles as we calculated for the returning early- and late-run adult fish (this assumes equal reproductive success and incubation/fry survival). To test for differences in the proportional relative productivity between the early run and late run, the estimated numbers of early- and late-run fry versus adults were compared using a crosstab chi-square analysis in 2000 and 2001 separately.

## Results

### Molecular genetic variation

High levels of microsatellite variation was observed in the adults (13–39 alleles; see Fillatre et al. 2003), and fry (9–45 alleles; Table 1). Significant deviations from Hardy–Weinberg equilibrium after Bonferroni correction were observed in eight out of 240 tests in the juvenile samples (Table 1). All deviations were due to a deficiency in heterozygotes, with the exception of site 5 in 2000 at locus Usat60. Significant deviations from Hardy–Weinberg equilibrium after Bonferroni correction were observed in 15 of 96 adult tests, and all were due to a deficiency in heterozygotes (see Fillatre et al. 2003). Significant differences were observed in the pairwise *F_ST_* comparisons of adult early run and late run (1999; F_ST_ = 0.023, *P* < 0.001; 2000 F_ST_ = 0.041, *P* < 0.001).

### Juvenile spatial distribution

Sockeye salmon fry were confined to Klukshu Lake and, to a much lesser extent, Klukshu River, based on extensive sampling of potential rearing habitats (Fig. 1). The observed source population divergence (*F_ST_*) combined with eight marker loci with an average of 12 alleles per locus per adult population (range 6–25 alleles) and approximately 50 individuals genotyped in each adult (source) population provides high assignment power (Cornuet et al. 1999). At the sites where fry were captured, overall fry assignment success (exclusion and rank-based) ranged from 56% to 85% in 2000 (average = 72%) and from 60% to 83% in 2001 (average = 73%). In total, 234 (out of 325) fry were successfully assigned in 2000, (46 early run and 188 late run), and 285 (out of 391) fry were identified in 2001 (75 early run and 206 late run).

**Figure 2:**
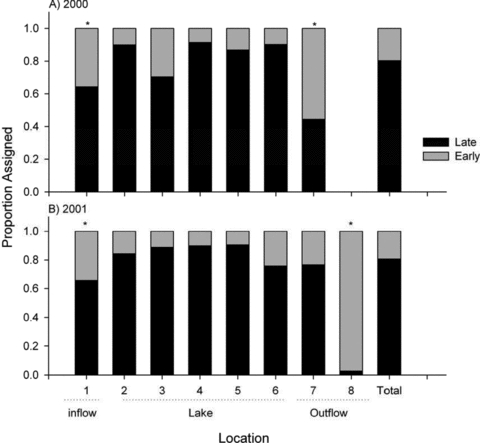
Relative proportions of sockeye fry assigned to the early run and late run that were captured in Klukshu Lake and River in 2000 (Panel A) and 2001 (Panel B) for the eight sites sampled (as described in Figure 1). The proportion of early- and late-run fry at each site was tested for departure from the total distribution across all sites using total count numbers and a crosstab chi-square analysis; asterisks denote significant departures (*P* < 0.05) from expected (i.e., sum total across all sites). In 2001, fry were captured in the Klukshu River (site 8) but not in 2000. We thus performed the analysis with and without the site 8 data, the significant departure of site 1 in 2001 from the expected distribution was only significant with the site 8 data excluded.

Both the early- and late-run fry utilize Klukshu Lake for rearing habitat, however the frequency of early- versus late-run fry differed significantly among the seven sites in 2000 (χ^2^ = 29.9, df = 6; *P* < 0.0001) but not in 2001 (excluding the Klukshu River site; χ^2^ = 10.7; df = 6; *P* = 0.098). When the Klukshu River site (site 8, Fig. 1) is included in the analysis for 2001, the frequency distributions are highly significantly different among sites (χ^2^ = 100.6; df = 7; *P* < 0.00001). At the individual site level, two sites showed significant divergence from the total early- and late-run fry proportions in 2000, and one site diverged in 2001 (Fig. 2; plus the Klukshu River site); in all three cases more early-run fry were found at the inflow and outflow sites than expected. At the Klukshu River site (site 8, Figs. 1 and 2), no fry were found in 2000, despite intensive sampling in the river. Only one location, site 7, showed a significantly different distribution of early- versus late-run fry between 2000 and 2001 (χ^2^ = 5.1; df = 1; *P* = 0.023), all other temporal comparisons showed no significant change in early/late composition at the individual sites (except the Klukshu River—site 8). We also used a log-linear model analysis to test for spatial and temporal differences in the proportion of early- and late-run fry: the log-linear model analysis agreed with the crosstab analysis results (see above) for all comparisons, although significance probability estimates were lower using the log-linear model.

### Adult relative productivity

**Figure 3:**
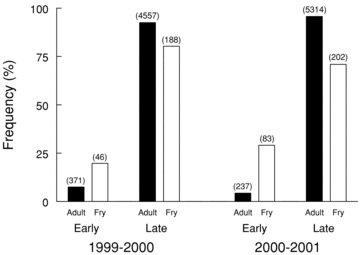
Frequency (and total numbers) of early- and late-run Klukshu River adult sockeye salmon in 1999 and 2000 (filled bars). Frequency (and numbers) of the offspring (fry) in the following year (i.e., 2000 and 2001, respectively) assigned to early- and late-run parentage (open bars). Crosstab analysis identified the early-run adults produce proportionally more fry than the late-run adults in both years (1999–2000: χ^2^ = 44.3; df = 1; *P* < 0.00001; 2000–2001: χ^2^ = 325.1; df = 1; *P* < 0.00001).

Relative productivity differed significantly between the early- versus late-run sockeye in both years (Fig. 3). In both years, a higher proportion of early-run offspring were produced than expected based on the numbers of returning adult spawners (2000, χ^2^ = 44.3; df = 1; *P* < 0.000001: 2001, χ^2^ = 323.1; df = 1; *P* < 0.000001) such that in 2000, 19.7% of the fry sampled were early-run offspring (while only 7.5% of the parental fish were early-run fish) and in 2001, 29.1% of the early-run fry were produced by only 4.3% of the adults identified as early-run fish. This means that the early-run adults produced 2.9 times more sockeye fry than the late-run adults in 2000, and 9.2 times more fry per adult spawner in 2001.

## Discussion

Understanding the distribution of biological diversity in space and time remains a recurrent challenge for conservation efforts, as the loss of intraspecific diversity may threaten the ability of species to respond to changing environmental conditions, and, ultimately, the stability and persistence of populations and fisheries (Hilborn et al. 2003; Schindler et al. 2010). Here, we employ molecular genetic and Bayesian assignment approaches to delineate the spatial distribution of allochronic populations of sockeye salmon during early life. Our results suggest allochronically isolated Klukshu Lake sockeye may utilize different habitats during early life, as 2000 late-run fry predominated in Klukshu Lake at all sites, while early-run fry were most numerous in the north and south (outlet) of Klukshu Lake. Moreover, the relative productivity ratios significantly differed, with the early-run population displaying significantly higher relative productivity. This study demonstrates the utility of genetic assignment methods to evaluate spatial partitioning and the relative contribution of allochronically isolated populations to species abundance and highlights the annual variability that may exist in both processes. The combination of molecular tools, assignment approaches, and life history information will be central to the successful conservation and maintenance of diverse adaptive portfolios in exploited species.

**Figure 4:**
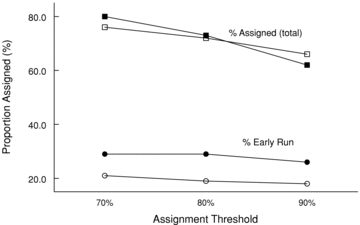
Results of the sensitivity analysis that varied the genotype assignment likelihood threshold from 70% to 90% (80% is used for the main analysis). Open symbols show results for 2000 fry, and filled symbols are for the 2001 fry.

The observed differences in juvenile sockeye salmon habitat use support the hypothesis that early- and late-run sockeye fry in Klukshu Lake partition their rearing habitat, at least early in the season. Similar differences in sympatric population juvenile habitat use have been reported elsewhere, such as in populations of juvenile dolly varden and bull trout in Thutade Lake, British Columbia, Canada (Hagen and Taylor 2001), juvenile Chinook and steelhead salmon in Bridge River, BC, Canada (Bradford and Higgins 2001), and juvenile sockeye and kokanee salmon in Takla Lake, BC, Canada (Wood et al. 1999). Although the exact mechanism driving habitat differences is unknown, a number of mechanisms for juvenile fish habitat partitioning in freshwater ecosystems have been proposed including clear habitat preferences by the fry and/or spawning adults, competition among individual fry, and increased survival and growth (Burgner and Rogers 1963; Pella and Burgner 1968; Seppä et al. 2001). For example, sockeye populations in Lake Aleknagik, Alaska have been shown to actively select preferred rearing habitats during the fry stage (Pella and Burgner 1968). Differences in juvenile distribution may result from differences in survival (Magurran et al. 1994; O'Connor et al. 2000), as observed in Arctic Charr, where populations composed of familiar individuals (those using similar environments during embryonic or early life development) showed increased survival, as compared with those in unfamiliar groups (Seppä et al. 2001). Competition between runs may also influence Klukshu Lake early- and late-run habitat partitioning; inter- and intraspecific competition related to changes in population density has been shown to influence habitat partitioning in other systems (Pella and Burgner 1968; Wood et al. 1999; Essington et al. 2000). Alternatively, juvenile habitat use may be correlated to parental spawning areas due to limited fry dispersal and thus their distribution reflects adult spawning site or timing divergence (Burgner and Rogers 1963; Wood et al. 1999). Indeed, the Klukshu early- and late-run sockeye are generally found near different spawning habitat as radio-telemetry has shown that the early-run adults are primarily found in Klukshu River (13/17 tagged fish), while the late-run fish tend to be found closer to lake beaches and shoals (16/20 tagged fish) (Petkovich 1999). However those data reflect fish distribution and not observed spawning, and over 20% of the adult fish had overlapping distributions. It is possible the year-to-year variation in early- versus late-run juvenile sockeye distribution may result from seasonal changes in habitat suitability; since we sampled the fry earlier in the season in the second year (July–August in 2000; June–July in 2001). Finally, there is the possibility that the fry assignment process itself may have introduced a bias into the proportion of early- versus late-run fry. To assess this, we replicated our analysis with both higher (90%) and lower (70%) assignment likelihood thresholds. Varying the assignment likelihood threshold does, as expected, change the number of fry successfully assigned (Fig. 4), but it has little effect on the relative proportion of sockeye fry assigned to the early run and late run (Fig. 4), nor was there any changes in the significance of any of the analyses associated with the assignment data. Thus, while we cannot rule out the possibility of assignment bias driving the observed patterns of juvenile habitat use, we feel it is an unlikely source of error.

This study demonstrates that genetic assignment coupled with extensive sampling provides a nondestructive means to estimate the relative productivity of the intermixed populations such as the early- and late-run Klukshu sockeye. Although genetic assignment is used routinely for mixed-stock assessment in fisheries science, our analysis is novel in that it compares the relative abundance of mixed-stock assigned fry to the known relative abundance of their returning parental populations. Surprisingly, in both years, despite lower numbers of early-run individuals, the early-run spawners exhibited a higher production of fry (relative to the number of spawners) than the late-run fish. Annual estimates of abundance indicate that the late-run fish are in decline (Fillatre et al. 2003) and as such this work supports the hypothesis that limitations in early life survival may be a contributing factor. Differences in egg production could influence fry abundance; however, although there is no fecundity data for the early run and late run, the two groups did not differ in body size (Fillatre et al. 2003). Thus, the observed difference in productivity is more likely due to variability in early life survival. Many factors may affect early survival, such as environmental conditions and food availability (Beacham and Murray 1987; Brannon 1987; West and Larkin 1987). Additionally, density-dependant effects may play a role in the elevated productivity of the early-run fish. Interestingly, if the relative proportion of early fry in 2000 and 2001 are compared to the relative proportion of early-run adult returning sockeye in 2004 and 2005 (the dominant return year for each of the fry study groups), we find close agreement. Early-run fry in 2000 comprise 19.7% of the fry, while returning early-run adults in 2004 comprise 22.9% of the run; and early-run fry in 2001 represent 29.1%, while returning early-run adults in 2005 represent 29.5%. Thus the two runs appear to survive at equivalent rates following their first year of life. Clearly more work needs to be done on the relative productivity of sympatric populations of fish, but this study does highlight the importance of the early life stages to survival and recruitment in Pacific salmon.

It is worth noting that both the early- and late-run fry utilize Klukshu Lake as their primary rearing habitat. No juvenile sockeye salmon were found in tributary environments (with the exception of the Klukshu River site in 2001), or in other smaller lakes in the system. Previous studies have reported fry from multiple populations of beach and stream spawning sockeye salmon within the same rearing lake, as well as sympatric populations of anadromous and nonanadromous sockeye salmon fry in the same rearing lake (Blair et al. 1993; Wood et al. 1999). Although multiple populations of salmon may use a common lake as a juvenile rearing habitat, it has not been clear from previous work if the offspring from the various populations are randomly distributed, or segregate to some degree within the lake environment.

Increasingly, support is building for the hypothesis that population diversity is directly associated with ecosystem stability and persistence (Tilman 1996; Schindler et al. 2010), though challenges remain for its quantification. Studies on sockeye salmon indicate that the stability of metapopulations and fisheries are enhanced through the maintenance of a variety of populations (Hilborn et al. 2003; Schindler et al. 2010), perhaps reflecting a diverse range of adaptations. We document differences in spatial distribution during the juvenile stage of allochronically isolated sockeye populations despite the fact that both are found in the lake environment. The factors responsible for these spatial differences remain unknown and require further study. Moreover significant differences in productivity and early life survival are consistent with adaptive differences among these early and late forms. Overall, this study demonstrates the important role of early life stages in the diversity and the maintenance of diverse adaptive portfolios.
